# Composition, Structure and Morphology Evolution of Octadecylamine (ODA)–Reduced Graphene Oxide and Its Dispersion Stability under Different Reaction Conditions

**DOI:** 10.3390/ma11091710

**Published:** 2018-09-13

**Authors:** Tianjiao Bao, Zhiyong Wang, Yan Zhao, Yan Wang, Xiaosu Yi

**Affiliations:** 1 Beijing Institute of Aeronautical Materials, Beijing 100095, China; bbttjj1234@126.com (T.B.); wy2011621@sohu.com (Y.W.); xiaosu.yi@biam.ac.cn (X.Y.); 2 Department of Materials Science and Engineering, Beihang University, Beijing 100191, China

**Keywords:** Octadecylamine (ODA), ODA–reduced graphene oxide, dispersity, reduction

## Abstract

Octadecylamine (ODA) can solve the aggregation problem of graphene sheets in the chemical exfoliation method. However, no attempts have been made to investigate the evolution of ODA–reduced graphene oxide (ORGO) with reaction conditions and the modification mechanism, which is the core problem to realize the controllable production and practical application of graphene. In this study, we treated graphene oxide (GO) with ODA under different reaction conditions to prepare ORGO. Fourier transform infrared spectroscopy (FTIR), X-ray photoelectron spectroscopy, atomic force microscopy, Raman spectroscopy, thermogravimetric analysis (TGA), and UV–vis spectrophotometry were employed to analyze the composition, structure, morphology and characteristics of the as–prepared graphene sheets. The results showed that the reduction reaction could occur under mild conditions, but the edge grafting reaction could only be activated by a higher temperature. Moreover, the ORGO created at 80 °C for 5 h and 120 °C for 0.5 h exhibited the optimized properties, both excellent dispersing stability and high heat resisting property, since they had more edge grafting chains and a suitable reduction degree.

## 1. Introduction

Since its excellent properties were widely reported [1], graphene and its derivatives were closely concerned in nanocomposite, conductors, and film material area [2,3,4,5,6]. Of key importance in the realization of graphene’s full potential are the development of bulk production methods and improvement of its dispersibility in non-aqueous solvent. Currently, chemical exfoliation [7] is the only method to implement quantity production and application of graphene. In chemical exfoliation, graphene oxide (GO) is prepared first, and then it can be reduced to reduced graphene oxide (RGO) by chemical [8] or thermal methods [9]. However, the as-prepared RGO is usually thermodynamically unstable due to strong intersheet van der Waals (vdWs) attractive forces and is hard to be exfoliated and stably dispersed in solvent. Much work [10,11] on exfoliation and dispersion of RGO in nonaqueous solvent was developed, since it may significantly facilitate the practical use of graphene, such as preparing nanocomposites, film materials and additives in lubricant. Adding stabilizers [12,13] is one method to improve the dispersion of graphene in solvent, but the presence of these stabilizers is not desirable for most applications. Another approach is to modify RGO by grafting, which usually has two steps: Grafting after the reduction or reduction after the grafting. For example, Ruoff et al. [14] functionalized GO with isocyanate to prevent aggregation in the reduction process with *N*,*N*-dimethylhydrazine. Nonpolar chemicals with long alkyl chains, such as octadecylamine (ODA), have been widely used to functionalize GO to improve its dispersion in nonaqueous solvent [15,16,17,18,19,20]. In previous works, ODA was mainly used as a surface modifier to improve the hydrophobicity of GO and its dispersion in non-polar polymers. For instance, Petridis et al. [16] reduced graphite oxide (GO) into graphite by either NaBH_4_ or hydroquinone, and then modified its surface with neutral, primary aliphatic amines and amino acids. Feng et al. [18] reported to reduce GO with hydrazine and then refluxed the reduced GO in a DMF solution of ODA at 90 °C for 48 h to improve its lipophilicity. Wang et al. [19] synthesized organophilic graphene nanosheets by modifying GO with ODA for 20 h, and then chemical reducing modified product with hydroquinone for another 22 h. Recently, Wenjuan Li et al. [21] demonstrated an approach to functionalize and in situ reduce GO with ODA without another reducing agent, which would simplify the process of producing solvent soluble RGO; this brought the promising future for the practical use of graphene. For practical application, as the additive for nanocomposite for example, the ideal structure of modified GO was both with high degree of reduction (for excellent mechanical properties, thermal properties and electrical properties) and excellent dispersing performance in non-aqueous solvent (for good dispersion in composite). It evoked our interest to know how to realize the ideal morphology, structure and properties of ODA modified RGO by controlling reaction conditions. As a consequence, more significant work should be completed to explore the mechanism of modification and trace the evolution of ODA–reduced graphene with reaction conditions, so that the structure of ORGO could be controllable and its properties could be designable. It was known to all that graphene’s final properties were significantly affected by various factors, such as defect, dimension, number of layers, grafting degree, reduction degree, etc. For example, it was easy to crease of the graphene with great dimension, which had a higher friction factor than the graphene with smaller dimensions. The graphitic structure’s higher degree of recovery could lead to better thermal property; however, the introduction of structure’s defects could exert a negative influence on the thermal property. In some circumstances, factors were closely connected and conflicted with each other. Therefore, we should thoroughly investigate the evolution of graphene with condition as well as the influencing and constrained relationships among various factors, understanding modification mechanism, so as to realize the design and control of materials’ properties. Furthermore, the treatment of GO with ODA in previous studies was usually under drastic conditions, i.e., conditions with high reaction temperature and long reaction time. Nevertheless, we expected to use a simple and high–efficiency process to produce graphene. As a result, we studied the structure, composition and morphological evolution of ODA–reduced GO (ORGO) in various conditions in our research. The dispersing performance and thermal properties were evaluated respectively to further comprehend and verify the evolution. Based on the study, we were able to obtain the desired RGO and realize its practical application by controlling conditions. In our study, we determined that a certain degree of reduction can occur at a low temperature (35 °C) in a short time (0.5 h), which can lower the production cost. Significantly, we identified the conditions to produce RGO with both high reductive degree and long–term dispersion stability. This result will facilitate the preparation of graphene–polymer composites and/or the development of graphene–based hybrid materials.

## 2. Materials and Methods

### 2.1. Raw Materials

GO was provided by Beijing institute of aeronautical materials, which was prepared by Hummer’s method. Octadecylamine (ODA), hydrazine hydrate (HHA, 85.0%), alcohol, tetrahydrofuran (THF), were analytical reagent grade and purchased from Sinopharm Chemical Reagent Co., Ltd. (Beijing, China).

### 2.2. Modification of GO

GO was prepared from graphite using the modified Hummer’s method [22], which was characterized by AFM (Bruker, Billerica, MA, USA), TEM and XRD in Figure S1 of Supplementary Materials. To be treated by modifier, 0.1 g of GO was added into 200 mL of absolute ethyl alcohol, and the GO/alcohol colloidal suspension was subjected to ultrasonication for 30 min. Then, 0.5 g of ODA was added into the solution with magnetic stirring and was refluxed under different reaction conditions. The prepared reduced graphene oxide (RGO) was washed 10 times by absolute ethyl alcohol together with suction filtration. At last, the filtered material was dried under vacuum (100 °C, 3 h). The ORGO film was obtained by filtration using a nylon membrane (0.04 μm, Whatman, Kent, England). The GO film was also prepared as the above method without adding ODA. The investigated reaction conditions were 35 °C, 0.5 h; 35 °C, 5 h; 35 °C, 24 h; 50 °C, 0.5 h; 50 °C, 5 h; 80 °C, 0.5 h; 80 °C, 5 h; 120 °C, 0.5 h; and 120 °C, 5 h, respectively. To analyze the structure and morphology of GO and RGOs, the samples of different conditions. were labelled. The GO was labeled as a, and ODA modified RGO with 35 °C, 0.5 h; 35 °C, 5 h; 35 °C, 24 h; 50 °C, 0.5 h; 50 °C, 5 h; 80 °C, 0.5 h; 80 °C, 5 h; 120 °C, 0.5 h; 120 °C, 5 h were named as b, c, d, e, f, g, h, and i, successively. All the characterizations of GO and RGOs in the result section were according to this order. The sample prepared at 35 °C for 24 h was only characterized by XPS to explain a certain conclusion, which was named as c-1. As a comparison, hydrazine hydrate (HHA) was used to replace ODA in the reduction reaction, and HHA reduced graphene oxide (HRGO) was prepared at 80 °C for 5 h, named as j.

### 2.3. Material Characterizations

General characterizations of the graphene oxide and reduced graphene oxide product were carried out by means of thermogravimetric analysis (TGA), X-ray photoelectron spectroscopy (XPS), Fourier transform infrared (FTIR) spectroscopy and Raman spectroscopy (Renishaw in Via, Gloucestershire, UK).

The original material GO was characterized by transmission electron microscope (TEM) using Tecnai G2 F20, FEI corporation (Hillsboro, OR, USA), and X-ray diffraction (XRD) using D8 advance (Bruker corporation, Billerica, MA, USA). The as–prepared reduced graphene oxide was characterized by atomic force microscopy (AFM) to show the particle size of the microscopic particle. The dispersion of RGO in tetrahydrofuran (THF) was observed after natural precipitation by digital pictures and UV–vis absorption spectroscopy (Shimadzu, Kyoto, Japan). XPS measurements were taken using a Thermo Escalab 250XI (Hillsboro, OR, USA) under 1 × 10^−10^ mbarwith monochromatic Al KR and an X-ray source with a power of 150 W. FTIR spectra were recorded with a Nicolet iS10 FTIR spectrometer (Hillsboro, OR, USA) using KBr pellets with a sample concentration of ~0.1 wt%. TGA was carried out using an SDTQ600 thermobalance (TA Instruments, New Castle, PA, USA) in air at a heating rate of 10 °C·min^−1^. UV–vis absorption spectra were recorded on a UV–2550 spectrophotometer (Shimadzu, Kyoto, Japan). AFM images were obtained using a Multimode8 instrument (Bruker Corporation) in a scan assist mode. Samples for the AFM imaging were prepared by drop–casting the dispersions onto freshly cleaved mica substrates (grade V–1, Electron Microscopy Sciences, Hatfield, PA, USA), which were then allowed to dry in air. Raman spectra were recorded using a Renishaw invia with a laser wavelength of 632.8 nm. The wettability of GO and rGO was tested by contact angle meter using DSA25 of Kruss Corporation (Wichita, KS, USA). Contact angle was measured right after dropping a 3 μL water droplet on each film.

## 3. Results

### 3.1. Composition and Chemical Structure of ORGOs

**Figure 1:**
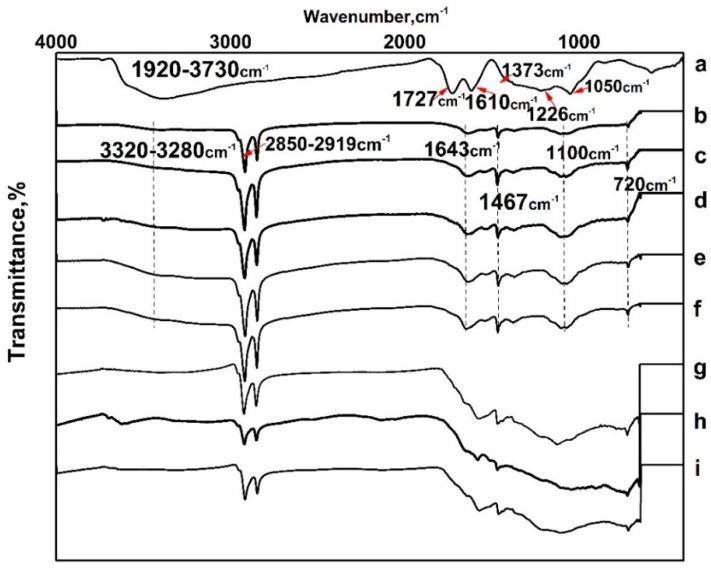
FTIR spectra of GO and ORGOs (**a**: GO; **b**: ORGO at 35 °C, 0.5 h; **c**: ORGO at 35 °C, 5 h; **d**: ORGO at 50 °C, 0.5 h; **e**: ORGO at 50 °C, 5 h; **f**: ORGO at 80 °C, 0.5 h; **g**: ORGO at 80 °C, 5 h; **h**: ORGO at 120 °C, 0.5 h; **i**: ORGO at 120 °C, 5 h).

**Figure 2:**
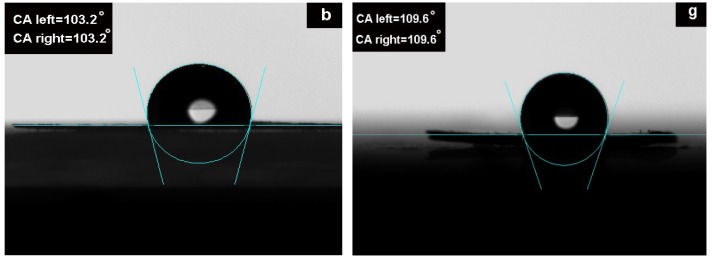
The water contact angle of typical ORGO ((**b**) ORGO at 35 °C, 0.5 h; (**g**) ORGO at 80 °C, 5 h).

**Scheme 1:**
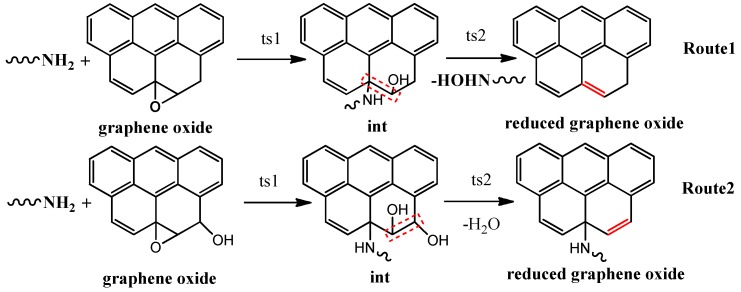
Deoxygenation mechanism of GO reduced by ODA.

The chemical changes occurring upon the treatment of GO with ODA can be observed by FTIR spectroscopy as both GO and its ODA–treated derivatives display characteristic IR spectra. The FTIR spectrum of GO (Figure 1a) was very typical [16,23,24]. The characteristic features in the FTIR spectrum of GO were the adsorption bands that correspond to the C=O bond of the carbonyl or carboxyl groups at 1727 cm^−1^, the O–H deformation vibration at 1373 cm^−1^, the C–OH stretching at 1226 cm^−1^, and the C–O stretching at 1050 cm^−1^. However, the FTIR spectra of ORGO were markedly different from that of GO. The signals of oxygen–containing functional groups (1727 cm^−1^, 1373 cm^−1^, 1226 cm^−1^, and 1050 cm^−1^) almost disappeared, which suggested the graphene oxide was reduced by ODA. Instead, the characteristic peaks of long–chain alkyls and amines appeared. As a reference, The FTIR spectrum of ODA was shown in Figure S2 (in Supplementary Materials). GO treated under mild conditions (Figure 1b–f) had similar characteristic peaks, i.e., bands at 3320–3280 cm^−1^, 2850–2919 cm^−1^, 1643 cm^−1^, 1467 cm^−1^, 1100 cm^−1^ and 720 cm^−1^, respectively. The single peak at 3280–3320 cm^−1^ is associated with the stretching of N–H of secondary amine. The characterized peak at these bands was totally different from multi-peak in spectrum of ODA which demonstrated the reaction between graphene oxide and ODA. The new bands at 2850–2919 cm^−1^ and 1467 cm^−1^ were the stretching and deformation vibrations of the C–H in methyl and methylene groups, respectively. The peak at 720 cm^−1^ was a characteristic peak of the linear connection in methyl groups. Furthermore, there was a new peak at 1100 cm^−1^, which was associated with the vibration of the C–N groups. This result was also confirmed by the XPS analysis and demonstrated the successful chemical reduction and surface grafting by ODA. The modification of GO by ODA was also reflected in Contact angle result (in Figure 2) indirectly. As we can see, after ODA functionalization, the removal of oxygenic group and the attaching of aliphatic chains made the ODA-GO film hydrophobic. Thus, the contact angle increased over 100 (as reported in previous study [20], the contact angle of GO was lower than 60). Considering that the reduction mechanism of hydrazine hydrate reduced graphene oxide in a former study [25] and the characteristics of primary amine groups, we speculated that the reduction occurred through two probable routes (shown in Scheme 1). In general, the reduction reaction was originated from nucleophilic addition between amine and epoxy in both the two routes. In a typical nucleophilic addition reaction, the amine group acted as a nucleophile and attacked the carbon atom in epoxy groups, grafting the long hydrocarbon chain of the ODA onto GO sheets. However, the deoxidation mechanism was different in route 1 and route 2. Route 1 was de–epoxidation process, in which the long–chain was exfoliation from graphene by reacting with hydroxyl at adjacent position and the deoxygenated product was then produced. On the other hand, when the epoxide group was adjacent to a hydroxyl group, the mechanism of route 2 might take place. In route 2, the epoxide ring was opened as well for the first step, while in the next step, two adjacent hydroxyls reacted and H_2_O was eliminated instead of the long–chain.

**Scheme 2:**
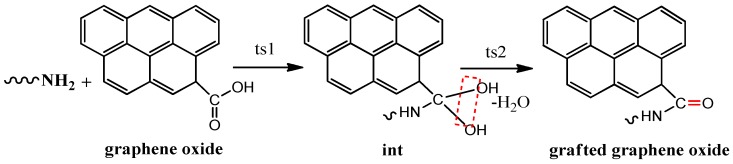
Reaction mechanism of ODA grafting onto GO.

The IR spectra of the RGO produced at a reaction temperature of 80 for 5 h or above 80 °C (Figure 1g–i) had completely different characteristic peaks. An absorption valley at 1000–1700 cm^−1^ appeared which was attributed to the association of the hydrogen bond in the amide group. Obviously, the amide was formed by the reaction of –COOH at the edge of GO with the –NH_2_ of ODA (named edge grafting). This indicated that the reaction of the edge carboxyl group could only be activated under certain conditions. That was why GO needed to be activated by sulfoxide chloride when ODA was used as a surfactant [25,26]. To give an explanation, the mechanism of edge grafting reaction was compared to that of reduction reaction. From Route 1 of Scheme 1 to Scheme 2, we figured that the two reactions all went through two steps, in which the first step was the nucleophilic addition reaction of oxygen containing groups and formed the same chemical bond. The only difference was that the epoxy ring was opened in the reduction reaction, while the carbonyl group was broken in the edge grafting reaction. Compared to the carbonyl group, the carbon–oxygen bond in epoxy has higher bond energy, so the activation energy of epoxy nucleophilic addition would be lower. According to the kinetics of chemical reaction, a higher temperature was needed to activate the reacting molecules to overcome the higher energy barrier (activation energy). Therefore, the edge grafting reaction needed a higher temperature to be activated.

**Figure 3:**
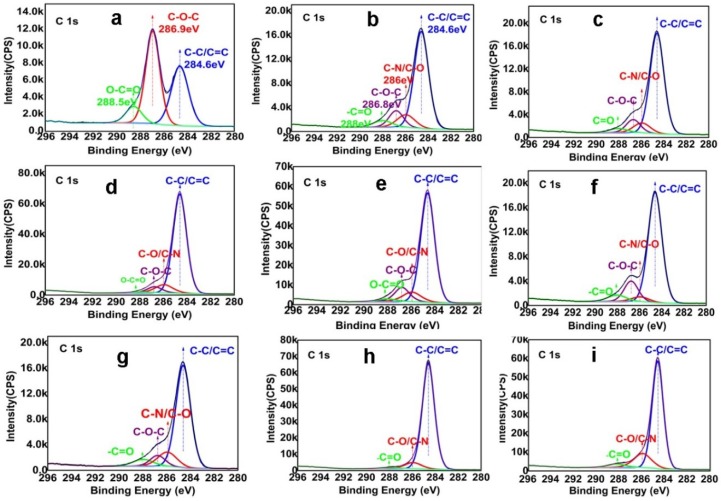
The deconvolution of the peaks in XPS C 1s spectroscopy of GO and RGOs ((**a**) GO; (**b**) ORGO at 35 °C, 0.5 h; (**c**) ORGO at 35 °C, 5 h; (**d**) ORGO at 50 °C, 0.5 h; (**e**) ORGO at 50 °C, 5 h; (**f**) ORGO at 80 °C, 0.5 h; (**g**) ORGO at 80 °C, 5 h; (**h**) ORGO at 120 °C, 0.5 h; (**i**) ORGO at 120 °C, 5 h).

An XPS analysis was performed to further investigate the reaction between GO and ODA, and the results were consistent with the IR spectra, which demonstrated the successful chemical reduction and surface modification of RGO. Figure 3 was the deconvolution of the peaks’ curves of XPS spectrum of GO and ORGOs. In brief, the C 1s XPS spectrum of GO had three components that corresponded to the carbon atoms in the different functional groups, i.e., the nonoxygenated ring C (284.5 eV), the C in the C–O or C–N bonds (286 eV), and the C in the C=O bonds (288 eV) [27,28]. Although the C 1s XPS spectrum of the ORGO also exhibited these same oxygen functionalities, the peak intensities were much less than those in GO, and the increase in the C–C/C=C signal was evidence of the graphitic carbon network restoration. This result confirmed the reduction reaction mentioned in the IR spectra analysis. Furthermore, to investigate the reductive degree of RGO under different reaction conditions, the ratio of different carbonate bonds was obtained by using the ratio of the bond peak areas in the XPS spectra (Table 1), and data of RGO treated by hydrazine hydrate (HRGO) and GO were used as comparisons (the deconvolution of the peaks’ curve of HRGO was shown in Figure S6 of Supplementary Materials). The results showed that the RGO created at 35 °C for 0.5 h already had a high ratio of C–C bonds, i.e., 70% from 39% of GO, and the reductive degree (refer to the percentage of C=C/C–C) slightly increased with higher temperatures and longer times (from 70% to 90%). This result was confirmed by TGA analysis and indicated that the reduction of GO by ODA could occur under mild conditions. Comprehensive performances of ORGO created at 35 °C for 0.5 h were shown in Figures S3 and S4 (Supplementary Materials). It proved that ORGO can be produced with ODA without heating, which was meaningful for quantity production. Moreover, the reductive degree of RGO at 35 °C for 24 h (its deconvolution of the peaks’ curve was added in Supplementary Materials) was close to that of the RGO created at 80 °C for 5 h, which was also comparable to that of HRGO (Table 1j). This revealed that the time and temperature were equivalent for the reduction of GO, and ODA has the same reduction efficiency as HHA under the same conditions. The time–temperature equivalent phenomenon was also discovered from the data of ORGO created at 80 °C for 5 h (Table 1g) and ORGO created at 120 °C for 0.5 h (Table 1h).

**Figure 4:**
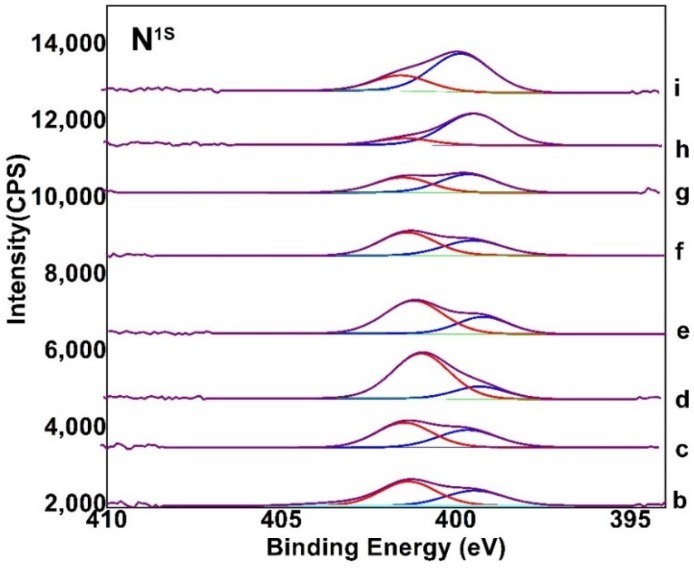
The deconvolution of the peaks in XPS N 1s spectroscopy of RGO (**b**: ORGO at 35 °C, 0.5 h; **c**: ORGO at 35 °C, 5 h; **d**: ORGO at 50 °C, 0.5 h; **e**: ORGO at 50 °C, 5 h; **f**: ORGO at 80 °C, 0.5 h; **g**: ORGO at 80 °C, 5 h; **h**: ORGO at 120 °C, 0.5 h; **i**: ORGO at 120 °C, 5 h), the red line was deconvolution peak of N–C at 399.5 eV and the blue line was N–H at 401.4 ev.

Signals at 399.5 eV and 401.4 ev corresponding to N–C and N–H appeared in N1s XPS spectrum of ORGO, which were not observed in the spectrum of GO. The deconvolution of the peaks’ curves is shown in Figure 4. The N–C signal represented the grafting effect of ODA onto GO, that is, the more grafting chains, the more intensive the N–C signal. The quantitative data in Table 2 showed that when the temperature was higher than 80 °C, there was an obvious increase of the N–C signal. These observations were consistent with the IR spectrum data (Figure 1.) and indicated that edge grafting occurs at 80 °C. The N–C signal also increased with a longer time (b to c-1 in Table 2), which agreed with the dispersion results. (The deconvolution of the peaks’ curve of c-1 was added in Supplementary Materials, Figure S5). As we speculated previously that the reduction reaction occurred in two steps: The ring-opening reaction occurred firstly, and the aliphatic chains attached onto the RGO in this step. Then the oxygenic group was reduced by eliminating amine or water. To obtain excellent dispersing performance, we expected to control the reaction condition, for instance by lowering the temperature or shortening the reaction time to keep it at the first step, so that the density of aliphatic chains on ORGO would be higher. However, the N–C signal was not stronger at low temperature or short time, and the reduction degree was high at mild condition. It pointed out that the deoxidation step occurred instantly as epoxide ring was opened even at low temperature, so that it was impossible to control the reduction reaction to suspend at the first step.

### 3.2. Morphology Evolution of ORGOs with Different Conditions

**Scheme 3:**
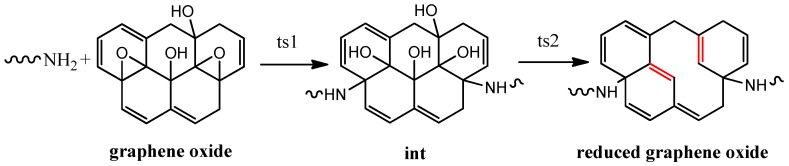
Mechanism of” Cut off “effect.

**Figure 5:**
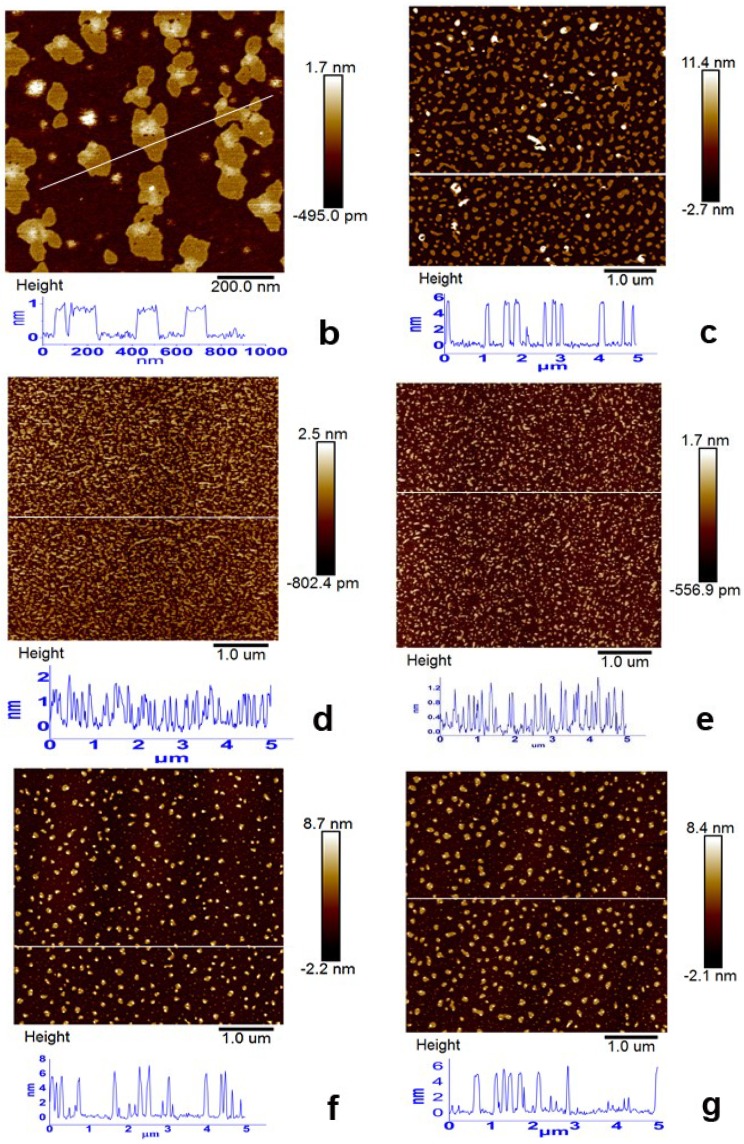

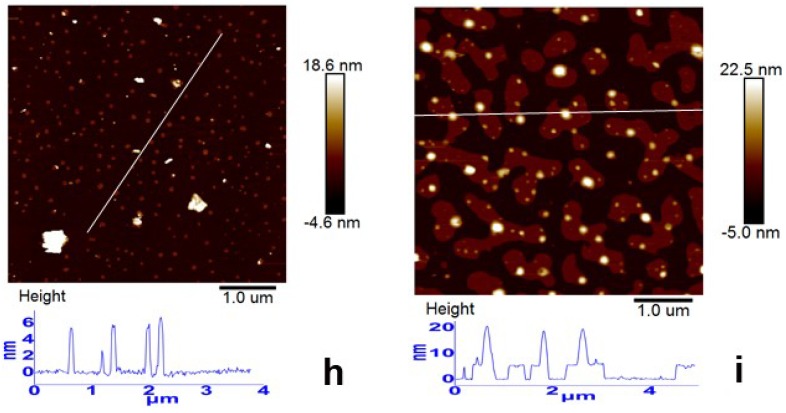
AFM image of RGO ((**b**) ORGO at 35 °C, 0.5 h; (**c**) ORGO at 35 °C, 5 h; (**d**) ORGO at 50 °C, 0.5 h; (**e**) ORGO at 50 °C, 5 h; (**f**) ORGO at 80 °C, 0.5 h; (**g**) ORGO at 80 °C, 5 h; (**h**) ORGO at 120 °C, 0.5 h; (**i**) ORGO at 120 °C, 5 h).

To investigate the degree of exfoliation and sheet dimensions of ORGO under different reaction conditions in THF, AFM imaging of the dispersions deposited onto mica substrates was carried out. Representative results were shown in Figure 5. The AFM images revealed the presence of irregularly shaped sheets with a uniform thickness that ranged from one nanometer to six nanometers, which indicated that ORGO can be exfoliated into one layer up to six–layer sheets in THF. This was important because most of the attractive properties of graphene and graphene–based sheets were mainly associated with their existence as individually separated entities. More significantly, after modification, the particle size of ORGO was greatly reduced compared to GO (refer to the TEM image of Figure S1 in Supplementary Materials). This phenomenon could be interpreted by the following speculation. The recovery reaction of new double bonds was accompanied with a large number of chemical bonds breaking, which had a “cut off” effect on the GO sheets and reduced the sheet dimensions. The probable mechanism of “cut off” effect was shown in Scheme 3. When elimination of H_2_O from two hydroxy happened at two adjacent carbon, the bond between them would break and form a defect. Then amounts of defects linked together, and the sheet was cut into little slices. It was obvious that the reduced size would also be beneficial for the stability of suspension.

**Figure 6:**
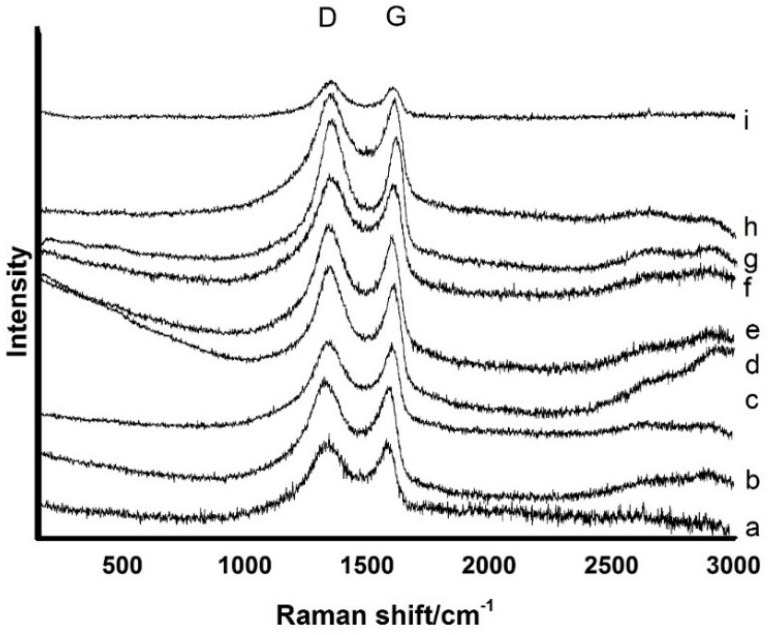
Raman spectroscopy of GO and RGO (**a**: GO; **b**: ORGO at 35 °C, 0.5 h; **c**: ORGO at 35 °C, 5 h; **d**: ORGO at 50 °C, 0.5 h; **e**: ORGO at 50 °C, 5 h; **f**: ORGO at 80 °C, 0.5 h; **g**: ORGO at 80 °C, 5 h; **h**: ORGO at 120 °C, 0.5 h; **i**: ORGO at 120 °C, 5 h).

Raman spectroscopy is a powerful, nondestructive tool that can be used to characterize carbonaceous materials, particularly distinguishing ordered and disordered crystal structures of carbon. Here, Raman measurements were performed, and the typical spectra are presented in Figure 6. Each spectrum showed three main peaks [29]. The G band at approximately 1580 cm^−1^ originated from the in–plane vibration of the sp^2^ carbon atoms and is a doubly degenerate phonon mode (E2g symmetry) at the Brillouin zone center. The 2D band (at approximately 2700 cm^−1^) originated from a two–phonon double resonance Raman process, and the D peak at approximately 1355 cm^−1^ was a breathing mode of the A1g symmetry that involved the phonons near the K zone boundary. The formation of defects was monitored by the ratio of the intensities of the D and G bands (I_D_/I_G_) in the Raman spectra, as shown in Table 3. The results showed that under mild reaction conditions (Figure 6b–f), the defects in RGO decreased since the restoration of the graphitic carbon network of the reduction reaction. The order degree of sample at intense condition (Figure 6g,h) was destroyed by long–chain grafting onto graphene of which I_D_/I_G_ as especially high. However, since the reductive degree of ORGO was incredibly high, created at 120 °C for 5 h, its I_D_/I_G_ was dropping.

**Scheme 4:**
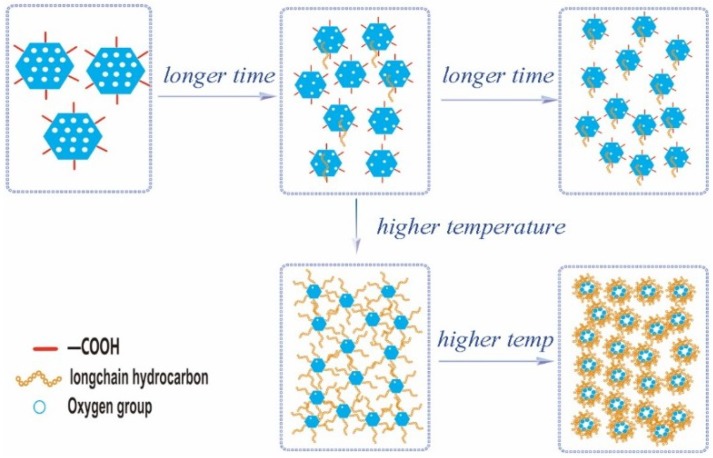
The evolution of RGO under different reaction conditions.

Ultimately, we constructed a model for the evolution of the composition, structure and morphology of RGO as the reaction times and reaction temperatures changed (Scheme 4). As we can see in Scheme 4, with a low temperature and short time, the reduction reaction occurred first; the oxygen–containing functional group significantly decreased with restoration of the graphitic structure. At the same time, some grafting occurred, and the particle size decreased. At this temperature, with a longer time more reduction reaction occurred; a slight decrease of the oxygen group content was shown, while the grafting chain had a slight increase. Considering the effect of the temperature, 80 °C was a demarcation point, i.e., below 80 °C, only the reduction reaction occurred, and above 80 °C, the reaction of edge grafting was activated. At 80 °C with a short time (0.5 h), the increase of grafting chain was still not obvious, since the reaction rate of edge grafting was still much lower than reduction reaction. The reduction degree also increased with the temperature, which finally resulted in the stacking and agglomeration of the RGO sheets at 120 °C for 5 h. This model could explain the exfoliation and dispersion result of different RGO dispersions.

### 3.3. Dispersion Stability of ORGOs

**Figure 7:**
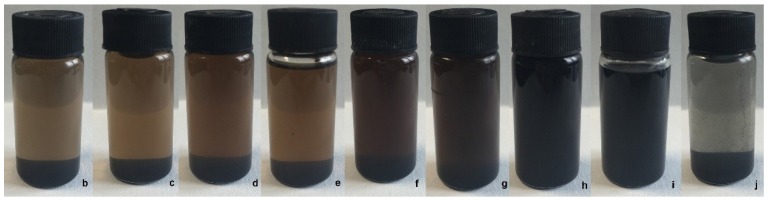
Digital pictures of ORGO and HRGO. ((**b**) ORGO at 35 °C, 0.5 h; (**c**) ORGO at 35 °C, 5 h; (**d**) ORGO at 50 °C, 0.5 h; (**e**) ORGO at 50 °C, 5 h; (**f**) ORGO at 80 °C, 0.5 h; (**g**) ORGO at 80 °C, 5 h; (**h**) ORGO at 120 °C, 0.5 h; (**i**) ORGO at 120 °C, 5 h; (**j**) HRGO, (80 °C, 5 h)).

As mentioned in the Experimental Section, the as–prepared reduced graphite oxide material was dispersed in THF at a nominal concentration of 2 mg·mL^−1^ with the aid of bath ultrasonication, and the dispersions were then allowed to settle for several weeks. Figure 7 shows digital pictures of the dispersions three weeks after sonication. For the just sonicated samples, all the reduced graphite oxide was dispersed in THF. However, many of these dispersions displayed only short–term stability and almost completely precipitated in a matter of hours or a few days (Figure 7b–d). In contrast, suspension of ORGO under intense conditions (Figure 7e–g) exhibited long–term stability, which showed a visually deep color. Although dispersion of ORGO at 120 °C for 5 h showed a deep color, the suspension was opaque with plenty of flocs.

**Figure 8:**
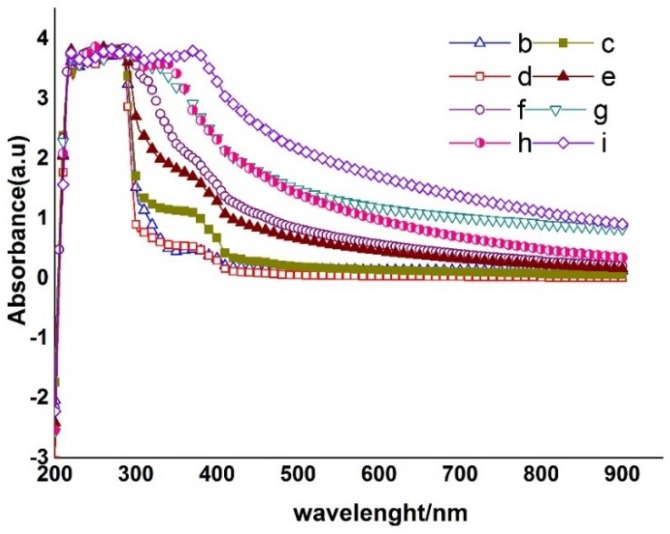
UV–vis absorption spectra of RGO and HGO (**b**: ORGO at 35 °C, 0.5 h; **c**: ORGO at 35 °C, 5 h; **d**: ORGO at 50 °C, 0.5 h; **e**: ORGO at 50 °C, 5 h; **f**: ORGO at 80 °C, 0.5 h; **g**: ORGO at 80 °C, 5 h; **h**: ORGO at 120 °C, 0.5 h; **i**: ORGO at 120 °C, 5 h).

Dispersion performance of ORGOs were also confirmed by the UV–vis absorption spectra. Figure 8 shows the UV–vis absorption spectra of RGO. The UV–vis spectrum of reduced graphene oxide exhibited two characteristic features that can be used for identification: A maximum at 231 nm, which corresponds to the π*π transitions of aromatic C=C bonds, and a shoulder at ~380 nm, which can be attributed to the n–π transitions of the C=O bonds [30]. Since the absorption peak at 200 nm was interfered by THF, while C=O bonds were stable under different reaction conditions, so we chose the absorption at 300–400 nm as a reference. For the different RGO samples, we noticed (Figure 8) that the RGO created at 120 °C for 5 h had the highest absorption intensity, but the suspending particles were flocs. This was closely followed by the curve changed, of the samples in Figure 8g,h, which exhibited very similar dispersion performance. At last, Figure 8b–f had notably poor dispersion stability.

It is generally acknowledged that GO could disperse in polar solvent like water and DMF [31]. On the other hand, GO had limited dispersing ability in medium polar solvent like THF, while the as-prepared ORGO could be easily dispersed in THF. Such dispersions are termed lyophobic colloids and often interpreted by the theory of Derjaguin, Landau, Verwey, and Overbeek (DLVO) [32]. In the DLVO theory of lyophobic colloids, there are three repulsive forces which can counteract attractive interparticle forces (vdWs in the case of graphene) and, if sufficient in magnitude, render dispersions stable for an extended period of time. These are (1) electrostatic, where the presence of surface charge establishes an electrical double layer (EDL) around the colloidal particles and results in an electrostatic repulsion between approaching particles, (2) steric, where bulky surface groups and solvent effect physically prevent the close approach of particles, and (3) Brownian movement, which improve the kinetics stability of lyophobic colloids. In our work, two key factors of grafting chains and reduction degree worked against each other resulting in the final dispersing properties of ORGOs. For instance, the grafting chains on ORGO increase steric hindrance physically, and improved the solvent effect according to Hildebrand and Hansen solubility parameters theory [33] (specific analysis was supplied in Supplementary Materials, Table S1). On the opposite, the high degree of reduction will increase the vdWs between different sheets, which breaks the stability of ORGO sheets in solvent. Additionally, the intense Brownian movement of ORGO with small particle size will improve the kinetics stability as assistant. It was not hard to understand why ORGO created at 80 °C for 5 h and 120 °C for 0.5 h has the best dispersion stability, since they had enlarged edge chains, and limited reduction degree. The samples created at temperatures lower than 80 °C had poor suspension stability, since they did not have enough grafting chains. For the sample created at 120 °C for 5 h, the dispersing ability was poor because of the strong Van der Waals’ forces due to the high degree of reduced graphene.

### 3.4. Heat Resistant Properties of ORGOs

**Figure 9:**
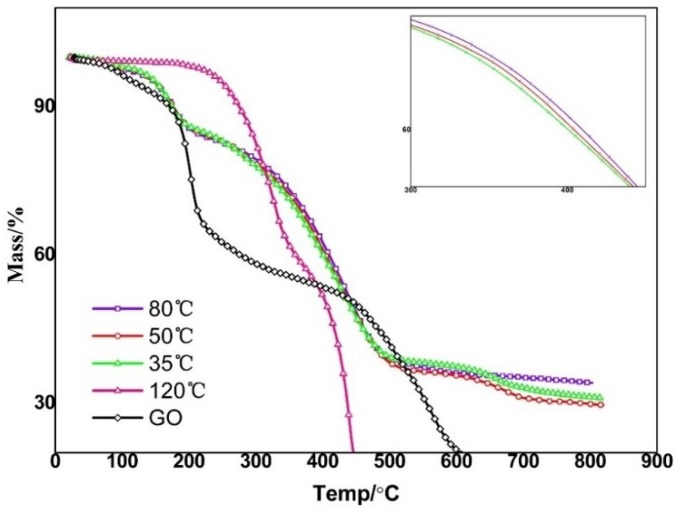
TGA of GO and RGO created at different temperatures.

**Figure 10:**
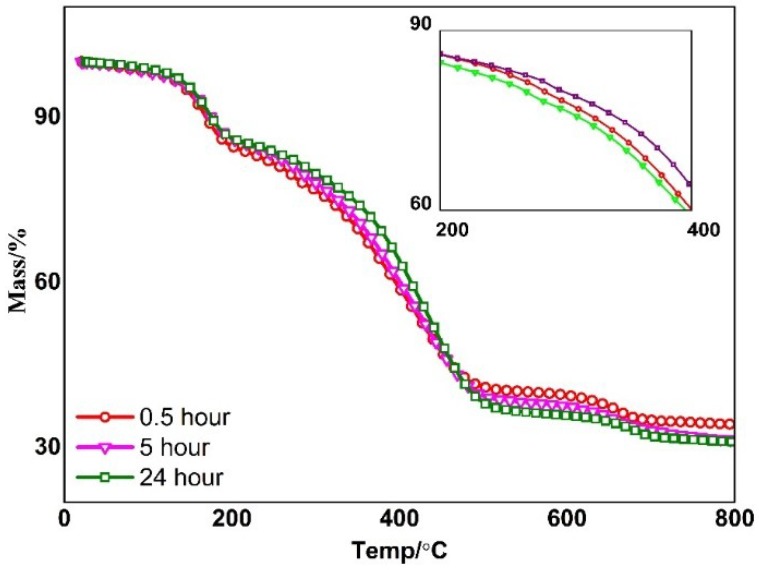
TGA of RGO created with different reaction times.

Thermogravimetric Analysis was performed to evaluate the structural recovery extent of ORGO compared to graphene. This characterization was necessary since all of the excellent properties of graphene were closely relevant to its graphitic structure. Figure 9 and Figure 10 presented the TGA curves for graphene oxide and reduced graphene oxide, which showed the mass loss during heating in air. Graphene oxide has mass losses in two temperature ranges. The first one, between 120 °C and 300 °C, corresponded to the removal of the oxygen–containing functional groups. The second one occurred at approximately 500 °C due to the bulk pyrolysis of the carbon skeleton [34]. The curves indicated that below 120 °C, the stability slightly increased with higher temperatures. The RGO created at 120 °C did not exhibit a substantial weight loss before 300 °C, while it had a drastic weight loss at 300 °C. The TGA performance was associated with the reduction degree and particle size. Since the reduction degree of RGO created at 120 °C was high, few oxygen–containing functional groups were removed before 300 °C. However, its particle size was smallest, which resulted in the drastic weight loss at 300 °C. The TGA results for RGO produced with different times and a certain temperature were shown in Figure 9. It was obvious that the thermostability slightly increased with longer times. These results agreed with the XPS spectra. The TGA result demonstrated that ORGO created at mild condition held almost the same heat resisted properties as ORGO created at higher temperature and longer time (Shown in Figure 10), which confirmed that the mild condition could be used to lower the cost of bulk production.

## 4. Summary and Conclusions

In conclusion, the reduction degree and grafting extent vary under different reaction conditions since the reaction mechanism changes. The reduction reaction initiated by the nucleophilic reaction between amine and epoxy group occurs easily, i.e., a high ratio of C–C signal was discovered in the XPS results at 35 °C for 0.5 h. The reduction degree slightly increased with a longer time and a higher temperature. However, the edge grafting reaction between amine and carboxyl group required intense condition. The evolution of the composition, structure and morphology of the RGO resulted in the different dispersion performances and heat resistant properties of RGO. The RGO created at 80 °C for 5 h and 120 °C for 0.5 h has the best dispersing stability since they have more edge grafting chains, small sheet size and suitable reduction degree. To sum up, the modification of ODA on GO can be controlled and the product can be designed based on the above conclusions. It is facilitated for the practical use of graphene: The high degree of reduction under mild condition (even closed to room temperature) was attractive for the industrial production of graphene-based composite/hybrid. The ORGO product with both a high reduced degree and a large number of grafting chains can disperse well in THF, and can prepare a resin-based composite with both high physical properties and tribological properties. Besides, the amine-modified GO could be used as an additive in lubricating oil and anti-corrosion additives in coatings. These investigative works. have been conducted and excellent results have been achieved. We will summarize and report them soon.

## Figures and Tables

**Table 1:** The aspect ratio of different C in the XPS C 1s spectra.

No.	Notes	C–O/C–N	C=O	C=C/C–C
a	GO	50	10	39
b	ORGO (35 °C, 0.5 h)	24	6	70
c	ORGO (35 °C, 5 h)	20	4	76
c-1	ORGO (35 °C, 24 h)	18	4	78
d	ORGO (50 °C, 0.5 h)	19	6	75
e	ORGO (50 °C, 5 h)	21	3	76
f	ORGO (80 °C, 0.5 h)	19	6	75
g	ORGO (80 °C, 5 h)	17	4	79
h	ORGO (120 °C, 0.5 h)	16	4	80
i	ORGO (120 °C, 5 h)	8	2	90
j	HRGO (80 °C, 5 h)	20	5	75

**Table 2:** The aspect ratio of different N in the XPS N 1s spectra.

No.	Notes	N–C	N–H
b	ORGO (35 °C, 0.5 h)	36.1	63.9
c	ORGO (35 °C, 5 h)	42.7	57.3
c-1	ORGO (35 °C, 24 h)	49.5	50.5
d	ORGO (50 °C, 0.5 h)	20.4	79.6
e	ORGO (50 °C, 5 h)	31.9	68.1
f	ORGO (80 °C, 0.5 h)	40.7	59.3
g	ORGO (80 °C, 5 h)	55.7	44.3
h	ORGO (120 °C, 0.5 h)	72	28
i	ORGO (120 °C, 5 h)	84	16

**Table 3:** Fitting parameters describing the I_D_/I_G_ for the ORGO shown in Figure 5.

No.	a	b	c	d	e	f	g	h	i
I_D_/I_G_	2.26	1.94	1.77	1.95	1.80	1.79	1.92	2.13	1.75

## Supplementary Materials

The following are available online at http://www.mdpi.com/1996-1944/11/9/1710/s1, Figure S1: The characterization of original material of GO (from left to right: XRD of GO; TEM of GO and AFM of GO), Figure S2: FTIR spectrum of ODA, Figure S3: TG of GO and RGO (35 °C, 0.5 h), Figure S4: AFM and digital picture of ORGO (35 °C, 0.5 h), Figure S5: Deconvolution of the peaks curves of C 1s and N 1s spectrum of ORGO created at 35 °C for 24 h, Figure S6: Deconvolution of the peaks curves of C 1s spectrum of HRGO (labelled j) created at 80 °C for 5 h, Table S1: Hansen Solubility Parameters for Various solvents.
