# Supplementary material for: Composition, Structure and Morphology Evolution of Octadecylamine (ODA)–Reduced Graphene Oxide and Its Dispersion Stability under Different Reaction Conditions
Source: Materials (Basel). 2018 Sep 13;11(9):1710. doi: 10.3390/ma11091710 (PMC6163944; doi:10.3390/ma11091710)
Supplement: Supplementary file 1 [file materials-11-01710-s001.docx]

Composition, Structure and Morphology Evolution of Octadecylamine (ODA)–Reduced Graphene Oxide and Its Dispersion Stability under Different Reaction Conditions

Tianjiao Bao ^1^, Zhiyong Wang ^1,^*, Yan Zhao ^2,^*, Yan Wang ^1^ and Xiaosu Yi ^1^

Supplementary materials:

**1. Characterization of GO**


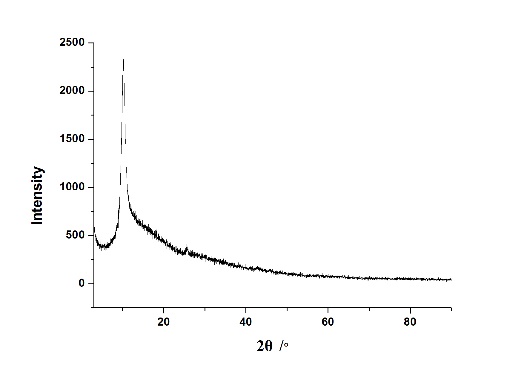

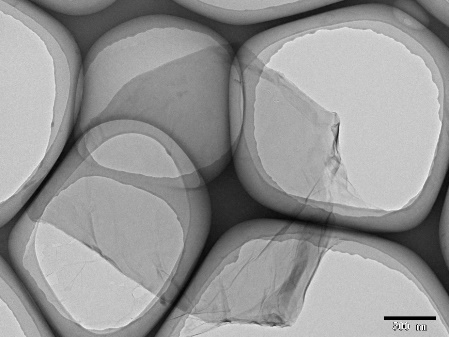

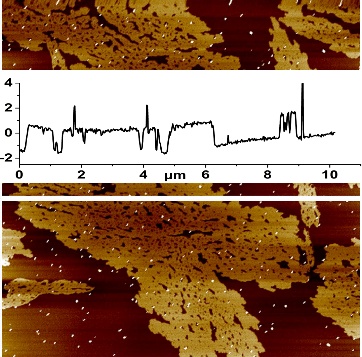


**Figure S1**. The characterization of original material of GO (from left to right: XRD of GO; TEM of GO and AFM of GO).

**2. FTIR of ODA**


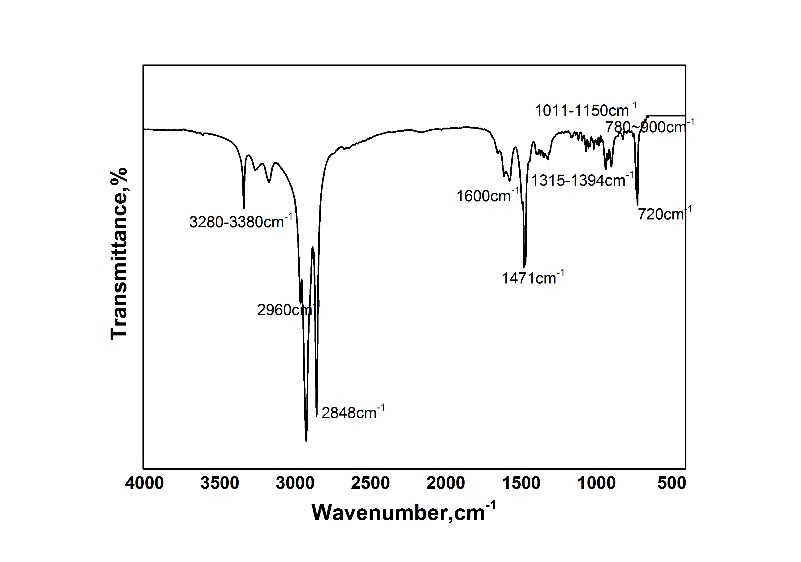


**Figure S2**. FTIR spectrum of ODA.

**3. Performance of ORGO at 35℃**

The RGO created at 35°C for 0.5 h already had a high ratio of C-C bonds, i.e., 70% from 39% in GO, which also had good overall performance of both high heat resistant property and well dispersity in THF. The TG curve of GO and ORGO showed that the heat resistant property was highly promoted of ORGO which could keep 70% of total weight at 360℃, while the GO has a significant weight loss at 220℃. The AFM images revealed the presence of irregularly shaped sheets with a uniform thickness of one nanometer, which indicated that ORGO can be exfoliated into one-layer sheets in THF. It also has well dispersity in THF of the just sonicated samples. For the sake of easily observation, the semitransparent dispersion of ORGO/THF with low density was prepared. As we can see from Fig. S3, the dispersion was uniform without any suspended particle which means that the ORGO was compatible with THF.





**Figure S3**. TG of GO and RGO (35℃,0.5h).

**Figure S4**. AFM and digital picture of ORGO (35℃,0.5h).

**4. Deconvolution of the peaks’ curves of XPS spectrum**


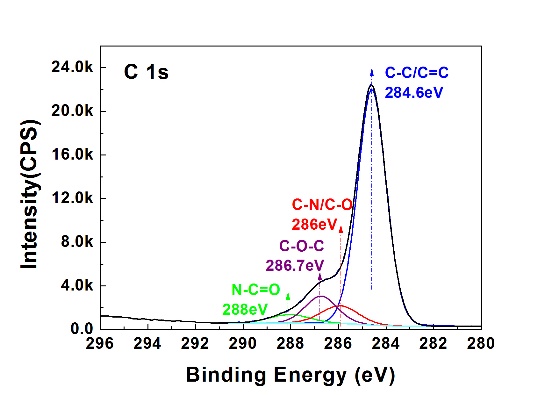

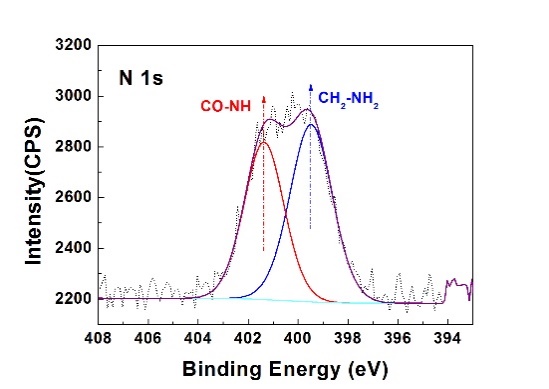


**Figure S5**. Deconvolution of the peaks’ curves of C 1s and N 1s spectrum of ORGO created at 35°C for 24h (labelled as c-1).


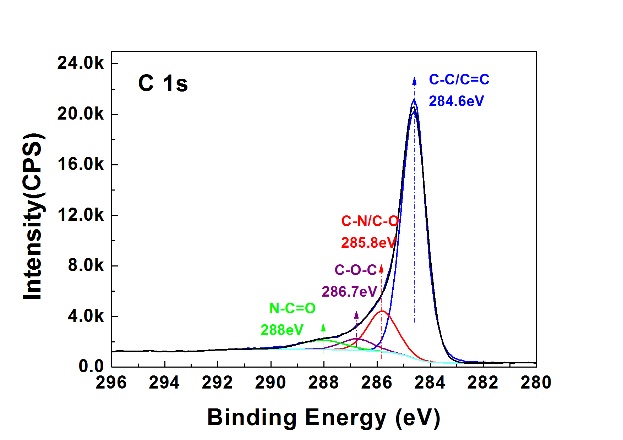


**Figure S6**. Deconvolution of the peaks’ curves of C 1s spectrum of HRGO (labelled j) created at 80°C for 5h.

**Analysis and discussion of soluble effect by Hansen’s theory**

According to the DLVO theory of lyophobic colloids, there are three repulsive forces which can counteract vdWs of graphene. These are (1) electrostatic, (2) steric, and (3) Brownian movement. The grafting chains on ORGO improved the solvent effect of steric force according to the Hildebrand and Hansen solubility parameters theory ^[33]^. The solvent effect meant that when the dispersion phase was compatible with the solvent, the solvent would form a solvent shell outside the particles, and the shell will prevent two particles from getting close. Hansen’s theory was usually used to predict the compatibility of the dispersion phase and the solvent. In brief, RGO and the solvent must both have similar Hildebrand solubility parameters and similar Hansen solubility parameters (HSP), with

| **$\text{δ}_{\text{T}}^{2}=\text{δ}_{\text{D}}^{2}+\text{δ}_{\text{P}}^{2}+\text{δ}_{\text{H}}^{2}$** | (1) |
| --- | --- |

where δ_T_ is the Hildebrand solubility parameter, and δ_D_, δ_P_, and δ_H_ are the Hansen solubility parameters corresponding, respectively, to dispersive, polar, and H-bonding components.

Several solvents were used to disperse ORGO, and their solubility parameters and dispersibility were listed in Table S1. As we know, suitable solvents for graphene oxide are characterized by δ_D_=17.4, δ_P_=13.7, and δ_H_=11.3(DMF). Dispersive component(δ_D_) was almost the same as these solvents, while the addition of alkyl chain and the reduction of graphene oxide resulted in the reduction of polar component(δ_P_), and H-bonding component (δ_H_), respectively (long-chain alkane like Dodecane as a reference). Hence, the as-prepared ORGO could easily disperse in solvents with medium polarity like THF, which has lower polar and H-bonding components than DMF. However, because of the partial grafting and reduction of ORGO, a nonpolar solvent like xylene was still not suitable for it.

**Table S1**. Hansen Solubility Parameters for Various solvents.

| No | Substances | δ_D_ | δ_P_ | δ_H_ | Dispersibility of ORGO |
| --- | --- | --- | --- | --- | --- |
| 1 | THF | 16.8 | 5.7 | 8 | YES |
| 2 | DMF | 17.4 | 13.7 | 11.3 | NO |
| 3 | xylene | 17.8 | 1 | 3.1 | NO |
| 4 | water | 15.6 | 16 | 42.3 | NO |
| 5 | Dodecane | 16 | 0 | 0 | —— |
